# Pressing Obligations or Inspiring Potentials? The Influence of the Ought vs. Expected Selves on Task Performance

**DOI:** 10.5964/ejop.v11i2.943

**Published:** 2015-05-29

**Authors:** Waclaw Bak, Slawomir Ciastek, Malgorzata Michalczuk

**Affiliations:** a Institute of Psychology, The John Paul II Catholic University of Lublin, Lublin, Poland; Aalborg University, Aalborg, Denmark

**Keywords:** expected self, ought self, obligations, potentials, task performance

## Abstract

This paper focuses on the effects of activating expected self as compared to the effects of activating the ought self. The expected self is a component of self-knowledge that pertains to the perception of one’s capabilities and potentials. Two experimental studies compared participants’ task performance after manipulating the momentary accessibility of the expected self vs. the ought self. In Study 1, contrary to expectations, the activation of the expected self resulted in poorer outcomes when the task required sustained attention. However, an interesting mood difference was revealed, which led us to hypothesise that activating the expected self results in slower (i.e., less hasty) work while performing the task. This hypothesis was confirmed in the second study.

## Introduction

Obligations are among the strongest regulators of human behaviour. All traditional societies place emphasis on the category of duties and responsibilities as important motivators for desired outcomes. Not only are they viewed as effective for motivating people to work on important society goals, but also as a means to reach one’s own personally relevant goals, especially those related to work, family and satisfying interpersonal relations. On the other hand, currently we can observe a tendency to deemphasise those categories of obligations, viewing them as a source of external motivation (see Deci & Ryan, 2008), which may even have detrimental effects on long term engagement and effectiveness of goal realisation. This is accompanied with the idea that every person has some positive potentials and that they function as resources which can be utilised as motivational strategies. Such an emphasis on personal possibilities and positive resources is employed in many domains such as coaching, management and business (e.g., Whitmore, 2009), education (e.g., Torp & Sage, 2002), and psychotherapy (e.g., González, Estrada, & O’Hanlon, 2011; Kita, 2011; O’Hanlon, 1998).

We do not claim conflict always exists between obligations and possibilities. A particular obligation may be congruent with one’s feelings of possibilities (e.g., the belief that the obligation is achievable). However at their core level, there seems to be a basic dichotomy between obligations and possibilities. The aim of the current studies were to examine which set of self-beliefs results in better task performance when activated. Is it better to focus on duty and responsibility or rather on one’s sense of having broad potentials and capabilities?

### The Ought Self vs. the Expected Self

In the present paper we address the above questions, assuming that they refer to the regulatory outcomes of activated self-knowledge. Two aspects of self-knowledge are crucial here. The first one refers to beliefs about what one should be like, which can be best conceptualised in terms of the *ought self*. The second aspect refers to beliefs about one’s possibilities, i.e. mental representation of what is attainable for the subject, what one believes he/she is capable of being like. This is best represented by the concept of the *expected self*.

The notion of the ought self is grounded in the self-discrepancy theory by Higgins (1987), who introduced two distinctions between domains of self-knowledge. First, there is a basic division between actual self and self-guides. Second, two types of self-guides are distinguished: the ideal self and the ought self. The ideal self is a cognitive representation of one’s hopes, aspirations and wishes for oneself, i.e. the attributes that one would ideally like to possess. The ought self in contrast is the representation of attributes that one believes one ought to possess referring to one’s duties, obligations and responsibilities. The basic idea of Higgins’ (1987) theory is that discrepancies between actual self and self-guides are distinctively connected to vulnerability for specific emotions. Discrepancy between actual self and ideal self relates to dejection related emotions, while discrepancy between actual self and ought self relates to agitation related emotions.

The second aspect of self-knowledge that we are interested in here is the expected self. It comprises the domain of self-knowledge that pertains to beliefs about one’s capabilities and potentials. The term expected self derives from Markus’ and Nurius’ (1986) influential theorising about *possible selves* (see also Hoyle & Sherrill, 2006; McElwee & Haugh, 2010). Possible selves are imaginations of future states of the self, referring to what is perceived as potentially possible for oneself. Among the different types of possible selves, the *expected self* is a possible self that it is not only possible to imagine, dream of, or think about, but one that is also expected to become the real self in the future. The expected selves are perceived by a subject as realistic and attainable, while the other types of possible selves (e.g., hoped-for ones) are more akin to loose aspirations for which attainability is not a crucial characteristic (Carver, Reynolds, & Scheier, 1994; Oyserman & Markus, 1990). The expected self may include both positive and negative possibilities (Sobh & Martin, 2008). There is, however, a kind of positive bias – a tendency to think about one’s potentials in positive rather than negative terms (Carver et al., 1994; Markus & Nurius, 1986).

### The Expected Self vs. Self-Efficacy Beliefs

The idea that self-beliefs regarding one’s capabilities and potentials play an important role in self-regulation has been clearly expressed by Bandura (e.g., Bandura, 2001) and widely studied within the self-efficacy paradigm (e.g., Bandura & Locke, 2003; Maddux & Volkmann, 2010; Wieber, Odenthal, & Gollwitzer, 2010). Thus, one can easily notice clear similarities between the concept of the expected self and the concept of self-efficacy. Indeed the connection between these two constructs was explicitly expressed by Oyserman and Markus (1990) in their seminal work on possible selves. When defining the construct (which includes the expected self as one of its domain) the authors stated that possible selves “are conceived of as the self-relevant, internal structures that embody and give rise to generalised feelings of self-efficacy” (Oyserman & Markus, 1990, p. 113). Nevertheless, there are some differences, both conceptual and methodological, between the approach employed in the studies presented below and the self-efficacy paradigm.

First, self-efficacy beliefs are conceptualised as operative capability – a belief that one can perform a given action. This operative aspect of self-beliefs, however, is not as central in the present study as it is in the case of self-efficacy. The expected self is the aspect of self-knowledge that may involve the belief that one can perform a particular action, however the agency is not crucial here. The expected self is a more general and less “agentic” belief that a particular self-view is possible to become the actual self in the future, no matter who is responsible for executing the outcome-resulting action.

Second, Bandura’s approach strongly emphasises the domain-specificity (or task-specificity) of self-efficacy beliefs. It explores how efficacy beliefs regarding a particular domain affect the effectiveness of dealing with tasks from this specific domain (Bandura, 2006, 2007; Maddux & Volkmann, 2010; Ozer & Bandura, 1990). We, by contrast, are interested in how the activation of specific self-beliefs affects performance in a domain that is not directly related to the content of the activated self-knowledge. The expected self in our studies was activated by asking the participants to generate lists of attributes, whose attainment lies within their capabilities. When listing their expected selves attributes, participants were fully free to choose their content. As a result, the specific contents of the activated expected selves differed across participants. However, the task performed afterwards was the same for everyone and was unlikely to be directly related to the activated self-knowledge of any participant, as in the case of domain-specific self-efficacy.

### The Present Research

The studies presented in this paper are based on the assumption that self-knowledge is not merely the description of one’s self-perception but dynamic knowledge that has an impact on emotions, motivation, and behaviour (Brown & McConnell, 2009; Higgins, 1996). Self-knowledge is a complex structure, composed of many different aspects. Depending on what aspect is currently active (accessible), different regulatory consequences arise (Higgins, 2000; Schlegel, Hicks, Arndt, & King, 2009). This phenomenon is usually addressed in experimental studies, in which changing the accessibility of a given aspect of self-knowledge is expected to result in a change in a person’s current functioning (e.g., Cesario, Grant, & Higgins, 2004; Liberman, Molden, Idson, & Higgins, 2001; Molden & Higgins, 2004).

The present research explored the regulatory consequences of making the expected self vs. the ought self momentarily accessible. Two experimental studies were conducted. The main hypothesis for the first study was that the activation of the expected self will have a more positive impact on task performance compared to the activation of the ought self. We assumed that the momentary accessibility of specific expected self attributes would activate a general belief in having broad capabilities and potentials. We further predicted that this general belief would translate into specific task performance, even if the task had nothing to do with the expected self attributes originally activated. We activated specific self-beliefs (a different set of expected self attributes for every person) but predicted a non-specific effect (the same task was administered to everyone). The belief that one has a wide range of resources and capabilities energises one’s motivation, which enables one to manage the task effectively. We expected that it would operate very much like optimistic beliefs about oneself (Carver, Scheier, & Segerstrom, 2010; Scheier, Carver, & Bridges, 1994). There are indeed empirically proven connections between the expected self and optimism (Carver et al., 1994). The ought self, in contrast, may activate extrinsic motivation, resulting in potentially detrimental effects on task performance (see Ryan & Deci, 2006).

We do not postulate, however, that activating the expected self improves the performance for every type of task. Our previous research suggests that these effects are less likely to be observed when performance depends mostly on such stable personal characteristics as general intelligence, particular abilities, or specific knowledge (Ciastek & Bąk, 2012). Still, even here some positive effects of activated expected selves may operate, though they refer to some additional aspects of performing the task, beyond its mere effectiveness. This is what the second study examined. The study tested the hypothesis that activating the expected self (compared to activating the ought self) will lead to a more accurate prediction of own future performance on a specific task.

## Study 1: Effectiveness of Task Performance

### Overview

The first study was designed to examine how the activation of the expected self (compared to the activation of the ought self) influences the effectiveness of task performance. A sustained attention task was used to measure the main dependent variable. Effective performance in such a task demands persistence in a long series of reactions. Although a single reaction was relatively easy and did not require any specific skills or abilities, the whole task could have been experienced as difficult in two ways. First, it lasted for a relatively long time and the participant did not know how long it would be. Second, the stimuli kept changing and getting more complex and more difficult in a manner that was difficult to predict. The participant did not know what to expect next and how long the whole task would last. Thus, we predicted that managing this task effectively requires believing in one’s capability to deal with unpredictable situations, i.e. a kind of efficacy beliefs (see Bandura, 2001). Given the conceptual similarities between self-efficacy and expected self, the activation of the expected self may lead to optimistic beliefs about one’s capacities to deal with unpredictability and uncertainty. This may strengthen the motivation to persevere in the task in the face of difficulties. Motivation, in turn, can improve attentional performance, as has been shown in several studies (e.g., Engelmann & Pessoa, 2007; Sänger & Wascher, 2011; Verhoeven et al., 2010). We predicted participants in the expected self condition not only to achieve better performance (Hypothesis 1) but also to be in a better mood after completing the task (Hypothesis 2).

### Method

Participants included 81 students (41 women) from four universities located in Lublin (Poland), representing different majors of study. Their ages ranged from 19 to 26 (*M* = 21.98; *SD* = 1.79). They were randomly assigned to two self-knowledge activation conditions (expected self vs. ought self) in a between-subjects design. The proportion of women and men was balanced between conditions. All participants received cinema ticket vouchers for their participation.

Participants signed up for individual 30-minute experimental sessions. After being greeted in the laboratory, each participant was seated in a private cubicle with a computer and two numbered envelopes on the desk. The envelopes contained paper forms to be used during the experiment. The entire procedure was programmed in *E-Prime 2.0* (Schneider, Eschman, & Zuccolotto, 2002). This included instructions concerning the use of the paper forms as well.

At the beginning, the researcher provided general information about the study and told participants that all detailed instructions would successively appear on the computer screen. Then he left the room and monitored the course of the experiment through a one-way mirror. After filling out basic demographic data (age, sex, and the field of study), the main elements of the procedure followed. It consisted of three stages: (1) the manipulation procedure, aimed at activating a particular component of self-knowledge, (2) the attentional task, and (3) the assessment of mood. At the end of the experimental session, participants were fully debriefed, thanked for their participation, and rewarded with cinema ticket vouchers.

#### Manipulations

Participants were randomly assigned to one of two conditions: the activation of the expected self vs. the activation of the ought self. In both conditions, the manipulation procedure consisted of two stages. First, participants generated a list of individual self-knowledge attributes (expected self vs. ought self). Second, they were asked to choose two attributes from the list they had just created and describe them in a more detailed narrative form. The two conditions differed in the aspect of self-knowledge that was explored. In the expected self condition, participants generated the list of expected self attributes in response to the following instructions:

“The task below concerns your beliefs regarding what you can become. It is about such positive traits and attributes whose attainment lies within your capabilities. Please devote the next 10 minutes to describing yourself as someone you really can become. Start by entering each positive possibility into a separate line below.”

Following these instructions, eight lines appeared starting with “I can …,” although there were no explicit instructions specifying how many attributes were to be generated. After filling in the list of attributes, the participant was asked to turn the page over and read the instructions for the second stage of manipulation:

“Now, from the list you have just created, choose two possibilities that you can describe in greater detail. (…). Then devote some attention to each of the possibilities chosen and describe it as if you wanted to tell someone about it in detail. Make sure your tale contains as many details and particulars as possible. Write what specifically this possibility is about, what it consists of, and what exactly you have in mind when saying that it is attainable.”

In the case of activating the ought self, the instructions were very similar except that they referred to “(…) your beliefs regarding what you ought to be like. (…) such traits and attributes that are connected with your sense of duty and responsibility”.

#### Attentional Task

After the manipulation form, a sustained attention task followed. Participants were asked to react when the letter “m” appeared on the computer screen. A total of 750 stimuli were presented one by one on the screen. Exposure time ranged from 500 to 750 ms, depending on the complexity of the stimulus. After each stimulus, a blank screen was presented for 300 ms and then the next stimulus followed. There were four types of stimuli: (a) single letters, (b) non-word strings of letters, ranging in length from three to nine letters, (c) correctly spelled words, and (d) incorrectly spelled quasi-words. They were presented in nine blocks as described in detail in Table 1. The blocks were presented in the same order to all participants, but the stimuli within each block were displayed in a random order, different for each participant. The correct reaction throughout the task was to press the designated key when the letter “m” appeared on the screen and refrain from pressing it when there was no “m” on the screen. The total number of correct reactions was calculated to assess task performance.

**Table 1:** The Details of Blocks of Stimuli Used in the Attentional Task

Block	Number of stimuli within the block	Exposure time for a single stimulus (ms)
1. Single letter	70	500
2. Non-word string composed of 3 letters	90	500
3. Non-word string composed of 5 letters	5	600
4. Non-word string composed of 9 letters	75	700
5. Non-word string composed of 7 letters	60	650
6. Mixed words and quasi-words. Series no. 1	100	750
7. Two non-word strings (one above the other)	100	750
8. Mixed words and quasi-words. Series no. 2	100	750
9. Mixed words and quasi-words. Series no. 3	150	750

The whole task lasted 12.5 minutes (the total exposure time for all 750 stimuli). However, participants knew neither how long it would be nor what type of stimulus would appear next. This was expected to bring about some kind of uncertainty and ambiguity for participants to deal with. The overall performance required persistence in sustaining attention for quite a long time. The additional requirement was the ability to deal with unknown situations. The task became increasingly complex and difficult, so the belief in one’s capability to deal with new situations was expected to be helpful.

#### Assessment of Mood

The UWIST Mood Adjective Checklist (UMACL; Matthews, Jones, & Chamberlain, 1990) was used to measure mood after the completion of the task. It assesses three dimensions of mood: hedonic tone, tense arousal, and energetic arousal. Hedonic tone refers to subjective feelings of pleasantness and relates to the dimension of positive affect. It is best depicted by such adjectives as: cheerful, contented, satisfied, happy, sad, sorry, and gloomy. Tense arousal is an anxiety related aspect of mood with highest loadings on: anxious, jittery, tense, nervous, calm, restful, relaxed, and composed. The third factor – energetic arousal – is best defined by such adjectives as: active, enthusiastic, excited, energetic, vigorous, unenterprising, sluggish, tired, and passive. Being in better mood (see Hypotheses 2) means greater hedonic tone, greater energetic arousal and less tense arousal. The reliability (Cronbach’s alpha) for the Polish adaptation of UMACL subscales ranges from .71 to .90 (Goryńska, 2005).

### Results and Discussion

We predicted that the activation of the expected self would lead to better performance in the attentional task compared to the activation of the ought self. Four outlying observations were removed from this analysis. The independent *t*-test revealed that the conditions differed in the mean number of correct responses (*t*(75) = -2.01; *p* = .048; *d* = .46), but the direction of this difference was opposite of what was expected. The mean for the ought self group was significantly higher (*M* = 701.70; *SD* = 15.99) than the mean for the expected self group (*M* = 691.20; *SD* = 27.99). Thus, the effectiveness of attentional processes was better in the ought self activation group.

The second hypothesis was that the participants with the expected self activated would be in a more positive mood after completing the task compared to the participants with the ought self activated. To verify this hypothesis, independent *t*-tests for UMACL subscales were calculated. Among the three dimensions of mood measured by the UMACL, the energetic arousal subscale differentiated between the experimental conditions and the direction of this significant difference was consistent with the hypothesis (see Table 2). Mean score on energetic arousal was higher for expected self group compared to ought self group. There were no differences between conditions in the other two dimensions of mood (hedonic tone and tense arousal).

**Table 2:** The t-Test for the Mood Variables (UMACL)

	Expected self		Ought self		*t*-test			
Variable	*M*	*SD*	*M*	*SD*	*t*	*df*	*p*	*Cohen’s d*
Hedonic tone	5.56	2.20	5.28	2.10	0.60	79	.55	0.13
Tense arousal	5.17	2.21	5.70	1.90	-1.15	79	.25	0.26
Energetic arousal	5.15	2.68	4.08	1.95	2.05	79	.04	0.46

Contrary to expectations, performance in the attentional task turned out to be better after ought self activation than after expected self activation. Activating positive beliefs regarding one’s capabilities and potentials did not have the predicted positive impact. In seeking an explanation for this result, we may hypothesise that the effects of expected self vs. ought self activation on task performance is moderated by the characteristics of the task. The results of this study do not preclude the possibility that expected self has some positive effects on task performance, however they may be limited to specific types of tasks. For other tasks, the ought self may have better impact, and the task used in our study seems to be a good example. Achieving a high score in the attentional task required that participants follow the instructions scrupulously and remain focused on the stimuli for a long period of time. What is important for such an activity is following the imposed external rules, staying focused on the instructions, and not questioning the reasonability of the work. Thus, activation of the ought self seems to fit these conditions better, as this aspect of self-knowledge refers to one’s sense of duty and responsibility.

The expected self, in contrast, is not concerned with obligations, but with capabilities and potentials. Therefore, participants in the expected self condition were not very pressed to achieve a good score in the task. As a result, their performance was in fact poorer compared to that of the participants in the ought self condition. We suggest, however, that this poorer performance was not caused by any kind of deficit or inability to focus attention but rather by being more relaxed and not regarding the task so seriously. They might have acted more calmly, with less haste, and not so much under the pressure of time. Such an interpretation seems to be consistent with the results referring to mood differences. Despite their poorer performance, after completing the task, participants in the expected self condition were more energetic, vigorous, enthusiastic, and at the same time – less tired and sluggish.

## Study 2: Accuracy of Predicting the Level of Performance and Pace of Solving the Task

### Overview

The second study went beyond the mere effectiveness of task performance. Regardless of whether there is an effect on the level of performance and irrespective of the direction of this effect, the activation of the expected self may affect some other aspects of coping with the task. This study tested the hypothesis that the activation of the expected self (compared to the activation of the ought self) will lead to a more accurate prediction of one’s own future performance on the task (Hypothesis 1) and a slower pace for solving the task (Hypothesis 2).

Prior to carrying out the task, participants were provided with general information about what they would be requested to do and then were asked to predict the expected level of their performance. The task used in this study required the ability to mentally manipulate and rotate objects, which is a relatively stable, individual difference variable from the domain of general intelligence (Matczak, Jaworowska, Ciechanowicz, & Stańczak, 2006). We did not expect then that the mean score of correctly solved trials would differ between experimental conditions. We did postulate however, that the activation of self-knowledge affects the accuracy of predicting one’s own future results.

In order to make such a prediction accurately, one needs to recognise both one’s own potentials and limitations. This aspect of self-knowledge is best represented by the concept of the expected self, so making the expected self momentarily accessible should improve the accuracy of prediction. Activating ought self, in contrast, may lead to either overestimating or underestimating the expected outcome of one’s upcoming activity. Overestimation is expected because the activated self-standard may result in focusing on reaching the highest quality, with the context of possibilities and personal constraints being somewhat ignored. Underestimation, on the other hand, may be a result of the discrepancy between actual self and self-standards. Activated discrepancy results in negative emotions and situational drop in self-esteem, which will probably result in lowered expectations regarding one’s own future performance.

The second study was designed to test this accuracy-of-prediction hypothesis. There was however, an additional hypothesis which stems from our interpretation of the results of the first study. We postulated that the activation of the expected self (compared to the activation of the ought self) would lead to a slower pace when performing the task. We assumed that this would result from less haste and less time pressure.

### Method

Participants included 81 students (41 women) from four universities located in Lublin (Poland), representing different majors of study. Their ages ranged from 18 to 26 (*M* = 21.99; *SD* = 1.79). They were randomly assigned to two self-knowledge activation conditions (expected self vs. ought self) in a between-subjects design. The proportion of women and men was balanced between conditions. All participants received cinema ticket vouchers for their participation. Although the sample has very similar characteristics as Study 1 sample, they are independent samples – a different set of people participated in Study 2.

Participants signed up for 30-minute individual experimental sessions. After being greeted in the laboratory, each participant was seated in a private cubicle with a computer and two numbered envelopes on the desk. The envelopes contained paper forms to be used during the experiment. The entire procedure was programmed in *E-Prime 2.0* (Schneider et al., 2002). This included instructions concerning the use of the paper forms as well.

The researcher started by providing general information about the study and telling participants that the detailed instructions would successively appear on the computer screen. Following this initial information, the researcher left the room and monitored the course of the experiment through a one-way mirror. After filling out basic demographic data (age, sex, and the field of study), the main elements of the procedure followed. It consisted of three stages: (1) manipulation procedure, aimed at the activation of a particular aspect of self-knowledge, (2) prediction of the level of own performance on the squares test, (3) performing the squares test. At the end of the experimental session, participants were fully debriefed, thanked for their participation, and rewarded with cinema ticket vouchers.

#### Manipulations

Participants were randomly assigned to one of two conditions: the activation of expected self vs. the activation of ought self. The manipulation procedure was very similar to that used in the first study. There was only one difference related to the expected self condition. In the first study, participants were asked to write down positive expected self attributes. In contrast, in the second study the instruction was to write down expected self attributes regardless of their valence. This modification is a result of the assumption that the accuracy of predicting one’s own upcoming performance is based on recognising both one’s own positives potentials as well as limitations. The instructions for the second stage of expected self manipulation (detailed description of the chosen two attributes) as well as the entire instruction for ought self activation were identical to Study 1.

#### The Squares Test

Following the manipulation, the participant returned to the computer and was provided with general information about the next task:

“In a moment, you will see 10 figures, one by one, on the screen. Each of these figures can be divided into two parts with a straight line and then reassembled into a square. The task will consist of finding, in each case, the fragment of the figure which should be cut off and put in a different place so as to make up a square. One minute will be allowed for solving each figure.”

Next, the sample figure was presented along with its correct solution. The participant was then informed that 10 figures will be presented, one by one, from the easiest one to the most difficult one. Before seeing the figures, participants were asked to predict their performance, i.e. to indicate how many figures they were expecting to solve correctly.

After making their predictions, participants were presented with the Squares Test. This task was adapted from the APIS-Z, which is a battery of general intelligence tests (Matczak et al., 2006). The test is designed to measure the ability to mentally manipulate and rotate objects. It consists of 10 irregular polygons. The task is to split each figure with a straight line into two parts in such a way that after rotation and reconnection they form a square. The original version of the squares test is a paper-and-pencil task, where all 10 figures are presented at once with a time limit of six minutes for solving the whole test. For the purpose of the present study, a modified version of the test was developed. In contrast to the original version, the figures were presented on the computer screen, one by one, from the easiest one to the most difficult one. The order of presentation was determined by the results of the APIS-Z standardisation study, which provided information about the difficulty level (the mean number of correct solutions in the standardisation sample) for each figure (Matczak et al., 2006).

Participants were informed that each figure would be presented for one minute. It was possible either to terminate the presentation earlier by pressing the specified key or let the presentation last for the entire time limit. If there was no action, the figure disappeared after one minute but it was still possible to write down the solution (on a paper report sheet). The next figure was not presented until the specified key had been pressed. The participant could then choose between approaching the next figure immediately after the previous one or taking a break, for which no time limit was specified. There were no explicit instructions either that the task should be done as quickly as possible or that it was possible to take a break between figures. Nevertheless, both the presentation time for each figure and the break time between figures were recorded for the purpose of verifying the second hypothesis. We assumed that they both reflect the pace of solving the figure and reveal whether a participant acted in haste. As there was no explicit instruction regarding the pace of doing the task, such haste would be self-imposed.

### Results and Discussion

As expected, there was no difference between the conditions in the mean number of correctly solved figures (*t*(79) = -0.78; *p* = .44; *d* = 0.18). The first hypothesis referred to differences in the accuracy of predicting the level of own performance. Accuracy was operationalised as the difference between the predicted and the obtained level of performance. Contrary to the expectations, the accuracy of prediction for expected self group (*M* = 2.32; *SD* = 1.68) did not differ from the accuracy for the ought self group (*M* = 2.63; *SD* = 1.90; *t*(77) = -0.16; *p* = .87; *d* = 0.04).

The second hypothesis was focused on the formal aspect of working on the task. More specifically, we hypothesised, that after being primed with the expected self, participants would work at a slower pace. Two analyses were conducted to verify this hypothesis. First, for each participant, we calculated the number of figures that were presented for the full one-minute period (i.e. figures whose presentation was not terminated intentionally by the participant). In order to meet the normality assumption for this variable we applied a square root transformation and removed two outlying observations. The independent *t*-test revealed a significant difference between the experimental conditions (*t*(71.99) = 2.31; *p* = .02; *d* = 0.52) and the direction of this difference was consistent with our prediction. There were, on average, more figures whose presentation was not interrupted intentionally by the participant in the expected self group (*M* = 1.87; *SD* = 0.68) in comparison to the ought self group (*M* = 1.45; *SD* = 0.91).

Consistent with this were the results of the second analysis. We measured the time spent on breaks between figures and calculated the mean time for each participant. One outlying observation was removed in order to normalise the distribution of this variable. The independent *t-*test revealed a significant difference between the conditions (*t*(69.40) = 3.03; *p* = .003; *d* = 0.67) and its direction is again in line with our hypothesis. Participants in the expected self condition took longer breaks between figures (*M* = 2762.66 (ms); *SD* = 1457.09) in comparison to participants in the ought self condition (*M* = 1931.94; *SD* = 955.32).

The results of both analyses were consistent and thus may be a manifestation of the same mechanism. After the expected self activation, participants were more likely (compared to the ought self condition) to utilise the whole time limit available for solving the task. They also took longer breaks between figures. One may say that participants in the expected self condition needed more time for solving the task correctly. There is, however, an alternative interpretation. The longer response times may indicate that participants in the expected self condition were calmer and felt less pressed for time when performing the task. In contrast, participants in the ought self condition worked in haste and under the pressure of time. What is important here is that this was self-imposed haste, since there were no explicit instructions saying that the task should be performed as quickly as possible or that it was better to note down the solution before the presentation of the figure ended.

## General Discussion

The presented studies focused on the regulatory consequences of making specific aspects of self-knowledge momentarily accessible. We were interested in situations when, prior to executing a specific task, a person focuses on his or her own attributes. Which aspect of activated self-knowledge has a better impact on task performance? Is it better to focus on beliefs regarding one’s capabilities and potentials (the expected self) or on beliefs concerning duties and responsibilities (the ought self)? We predicted the former rather than the latter focus would improve performance and we tested this hypothesised positive effect of activating the expected self in two studies. The results showed that focusing on the expected self may have different effects than focusing on the ought self. Nevertheless, the nature of this difference was complex and seems to depend on a specific aspect of performing the task.

The first study did not reveal the predicted positive effect of activating the expected self on the level of task performance. The results were just the opposite. Performance on the attentional task was better when participants were focused on their duties, obligations, and responsibilities. We argue that this effect might have been caused by the fact that this task required following instructions scrupulously and being focused on relatively boring stimuli for a long period of time. Following such imposed, external rules seems to fit the ought self condition better, since this is the aspect of self-knowledge that explicitly refers to duty and responsibility (Higgins, 1987, 1997). People in the ought mind-set probably worked harder, because they felt influenced by social expectations regarding their performance. The results of our study do not preclude the possibility that for some other types of tasks activating the expected self may improve performance. It suggests, however, that when a task requires being focused on the instructions and doing some relatively boring job the ought self (as compared to the expected self) has better impact on task performance. It seems that the ought self rather than the expected self should be activated when one wants to achieve a high level of performance on such tasks.

Regardless of the above, we have additional results suggesting that certain positive effects of making the expected self accessible do in fact occur. In the case of the first study, we found that, regardless of their poorer performance, the participants in the expected self condition were more energetic after completing the task. Consistent with this were the results of the second study, which were not focused on the level of performance itself, but on some additional formal aspects of performing the task. This time we did not expect differences in the effectiveness of performance, and there were no differences indeed. However, we also did not find a difference, which we did postulate – one which referred to the accuracy of predicting the level of performance. On the other hand, in line with our predictions, participants in the expected self condition worked slower while performing the task. They did not rush as much as the ought self participants did, yet reached the same level of performance. We suggest that this means working more calmly, with less haste, and not being so susceptible to time pressure.

In conclusion, we can state that even if activating the expected self does not improve the effectiveness of task performance, it seems to positively impact the way a task is performed. It probably creates favourable conditions for feeling better when doing the work and immediately after the work has been done. It also promotes being more relaxed and not so prone to external pressure to hurry up. In some cases, it may result in poorer achievement. In others, it does not affect the level of performance. Still, this does not preclude the possibility that for some other types of tasks the expected self improves performance. Future studies should explore this third possibility by looking for the specific characteristics of such tasks. In light of the two studies presented in this paper, we cannot say that “Yes, We Can” is always the best motivator. Nevertheless, there are situations, in which activating this potential-focused state of mind may still be a good idea.
